# Supplementary material for: Experimental demonstration of angular momentum-dependent topological transport using a transmission line network
Source: Nat Commun. 2019 Jan 25;10:434. doi: 10.1038/s41467-018-08281-9 (PMC6347663; doi:10.1038/s41467-018-08281-9)
Supplement: Supplementary file 1 — Supplementary Information [file 41467_2018_8281_MOESM1_ESM.pdf]

Supplementary Information of

"Experimental demonstration of angular momentum-dependent topological transport using a transmission line network"

Jiang et al.

Supplementary Information of

"Experimental demonstration of angular momentum-dependent topological transport using a transmission line network"

Tianshu Jiang[+], Meng Xiao[+], Wen-Jie Chen, Lechen Yang, Yawen Fang, Wing Yim Tam and C. T. Chan[*]

Department of Physics, Hong Kong University of Science and Technology, Clear Water Bay, Hong Kong, China

[+]These authors contributed equally to this work.

[*] Correspondence address: phchan@ust.hk

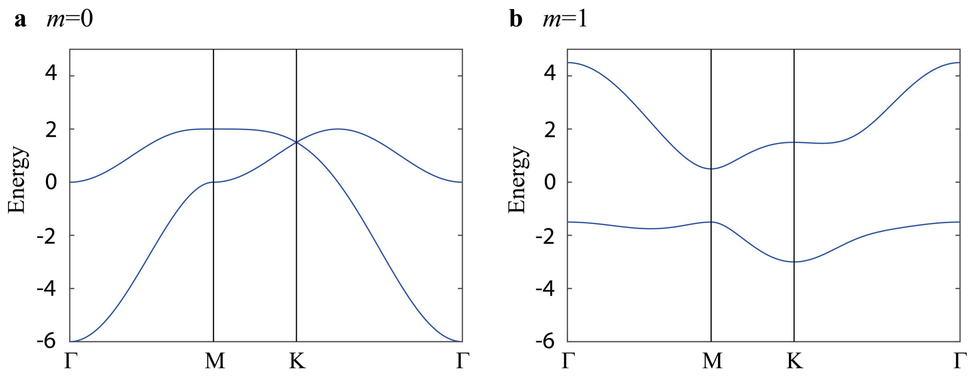

Supplementary Figure 1| Band structure of the tight-binding model. (a, b) The bulk bands for *m*=0 (a) and *m*=1 (b). The band structure for $m=-1$ is the same as that in (b). In this calculation, $t_1=-0.5$ and $t_2=-1$.

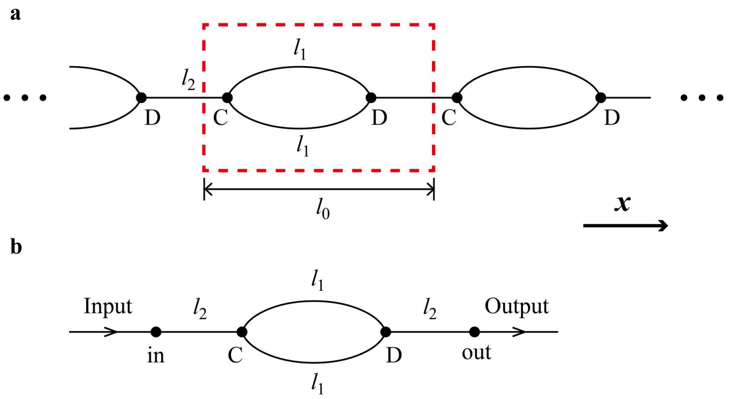

Supplementary Figure 2| Examples for the calculations in the transmission line network. (a) A one dimensional periodic transmission line network. (b) A primary network with an open boundary.

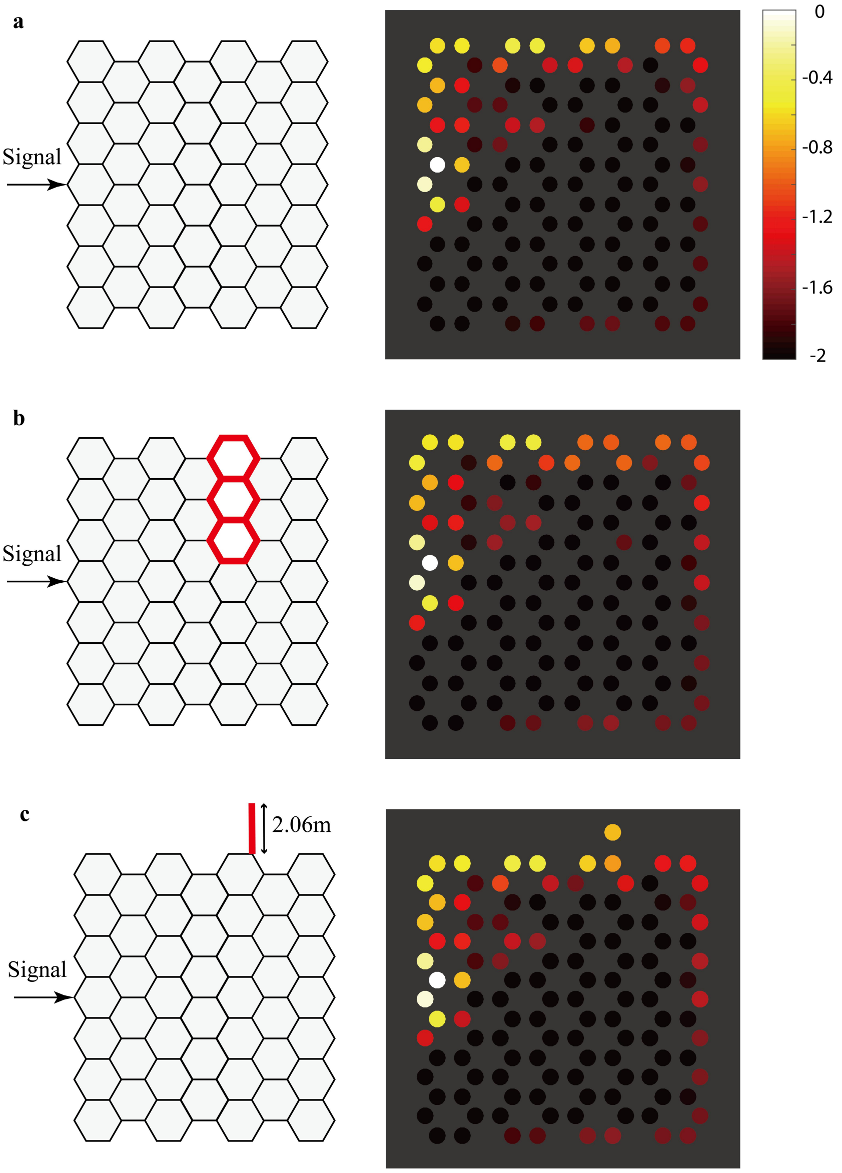

Supplementary Figure 3| Simulated edge state excitations. Left panels show the network configuration while right panels show the simulated field distribution. The color of each node represents the value of $\ln\left|U/U_{\max}\right|$, where U is the voltage at each node, and $U_{\max}$ is the maximum voltage among all nodes. (a) A finite-size sample without defect. (b) The same sample but with all the interlayer cables removed for the unit cells highlighted in red. (c) The same sample with additional cables (2.06 m in length) at the upper edge (highlighted in red). In all three cases, edge waves pass through the defect and propagate forward without back-scattering. In all simulations, we set m=1 and the working frequency to 32.7 MHz which is inside the nontrivial band gap.

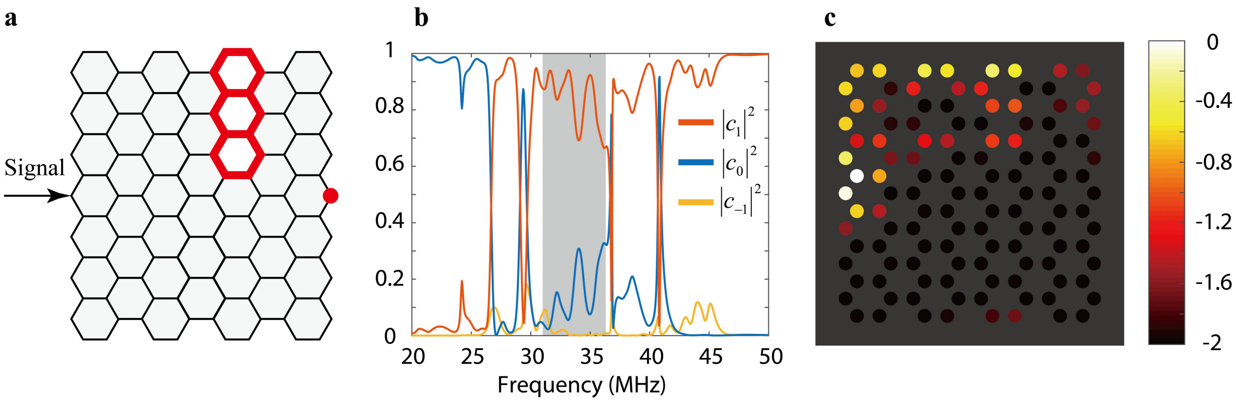

Supplementary Figure 4| A defect that does not conserve angular momentum. (a) The schematic of a network with angular momentum non-preserving defect which is introduced by removing the interlayer cables between layer 1 and layer 2 in the red unit cells and the interlayer cables between other layers are kept. Note that in the defect configuration shown in Supplementary Fig. 3(b), ALL interlayer connection cables are removed in the red unit cells, and the defect does not destroy angular momentum conservation. Here, only the cables between layer 1 and 2 are removed. As such, the defect is angular momentum non-conserving. The signal bearing angular momentum $m = 1$ is incident from the point marked by the black arrow. (b) shows the simulated transmission spectra of the coefficients $|c_1|^2$, $|c_0|^2$ and $|c_{-1}|^2$ at the point marked by red in (a), where c_i denotes the transmission coefficient with angular momentum m=i, and gray marks the non-trivial band gap region of $m = \pm 1$. (c) The simulated field pattern on the second layer at 32.7 MHz for the network in (a). Compared with Supplementary Fig. 3(b), the edge state is now scattered into other angular momentum states, in particular the m=0 state. This is because the angular momentum is no longer conserved. Note that the band gap highlighted in gray color is for $m = \pm 1$. The subspace with $m = 0$ exhibits no band gap in that frequency and hence the edge wave can be scattered into bulk, as shown in the figure.

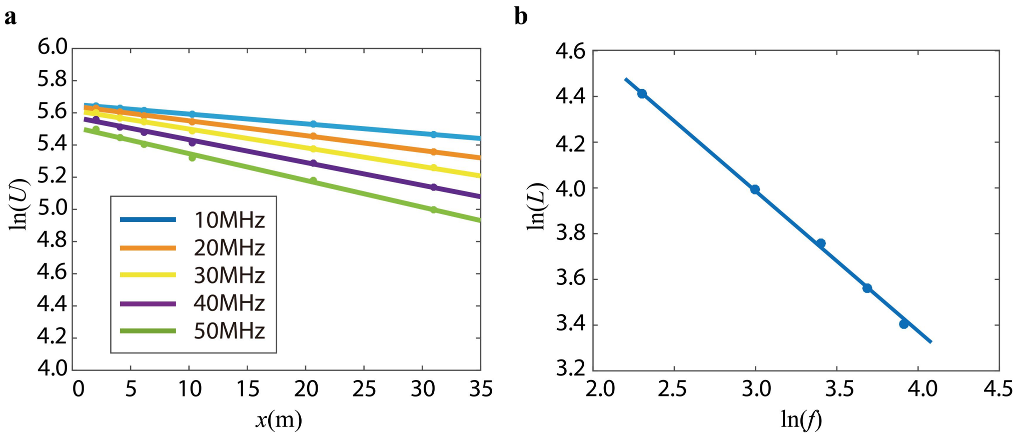

Supplementary Figure 5| Absorption length of our cable. (a) Linear fitting of ln(U) as a function of x for different frequencies, where x is the cable length and U is the measured wave amplitude. The absorption length can be obtained from the slopes of the fitting lines. (b) Linear fitting of the ln(L) with ln(f) according to the empirical relation $L \sim \alpha f^{-\beta}$, where L is the absorption length in meters and f is the frequency in MHz. In (a) and (b), the dots represent data points and the curves are from numerical fitting.

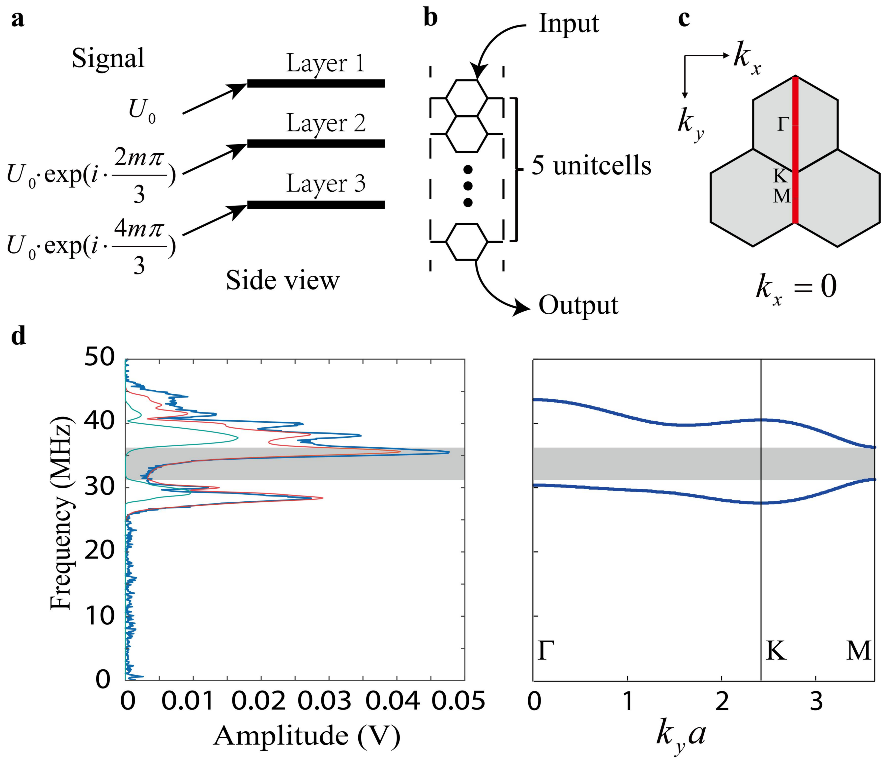

Supplementary Figure 6| Excitation of states carrying a specific angular momentum and directional band gap. (a) This panel illustrates how to excite the mode with a specific angular momentum. A side view is shown. Nodes at the same position in different layers are excited with the same amplitude and the corresponding phase difference. (b) Measurement of the bulk transmission along the Γ-K direction. The picture shows a top view of the strip and periodic boundary conditions are imposed in the *x* direction. In the strip, there are five unit cells aligned in the *y* direction in our measurement. (c) The red line in the Brillouin zone highlights $k_x = 0$ in the reciprocal space. (d) The transmission spectrum (left panel) and bulk band (right panel) for *m*=1. Gray color represents the bulk gap region. In the left panel, the blue curve represents the experimentally measured transmission spectrum and the red curve represents the numerically calculated spectrum with the same setup and an extra length of 0.06 m added to each cable. The cyan curve shows the transmission spectrum with 20 unit cells along the *y* direction and other parameters are kept the same as those for the red curve. To see the transmission spectrum clearly, we magnify the relevant section of the cyan curve by five times.

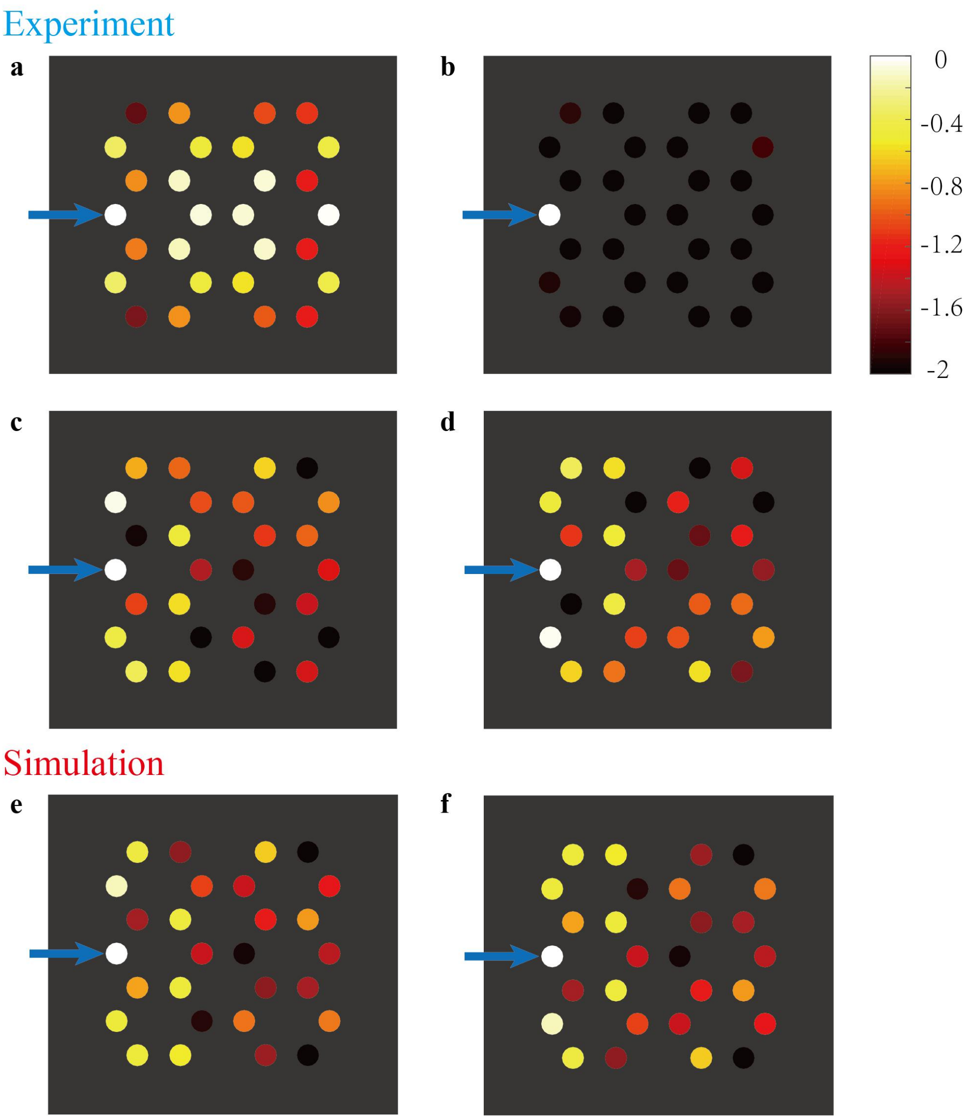

Supplementary Figure 7| Field patterns of propagating and non-propagating states. (a)-(b) The experimentally measured field patterns at 33.7 MHz (a) and 49.2 MHz (b) for m=0. (c) and (d) show the experimentally measured field patterns at 40.3 MHz for $m=1$ and $m=-1$, respectively. (e) and (f) show the corresponding numerical simulation of the experimentally measured patterns in (c) and (d), respectively. In (a)-(f), the positions of sources are the same as marked by the blue arrows.

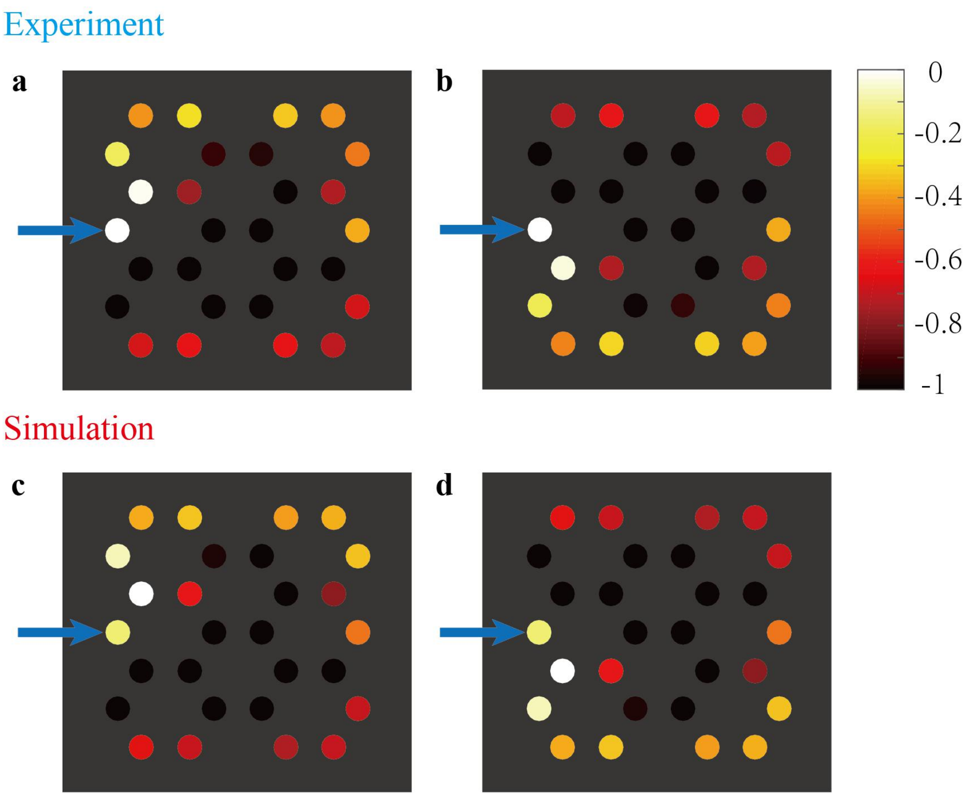

Supplementary Figure 8| Experimentally measured and numerically simulated voltage magnitude distribution inside the nontrivial gap. (a)-(b) show the experimentally measured field patterns at 31.2 MHz for $m=1$ and $m=-1$, respectively. This frequency falls inside the nontrivial gap, and hence we can observe the edge state field patterns. The propagation direction is locked to the sign of *m*. (c)-(d) show the simulated field patterns at 31.2 MHz for $m=1$ and $m=-1$, respectively. The simulation results agree well with the experimental results.

Supplementary Note 1: Hamiltonian of the tight-binding model

For the model shown in Fig. 1(g), we label the nodes as A_i (B_i), where A (B) denotes the sublattice and i denotes the layer number successively from top to bottom. Take N=3 as an example. We will show that there exists an effective angular-momentum-orbit coupling in our system. In the momentum space, the Hamiltonian H of our tight-binding model under the basis $(|A_1\rangle,|B_1\rangle,|A_2\rangle,|B_2\rangle,|A_3\rangle,|B_3\rangle)$ can be written as

$$H(\mathbf{k})=\begin{pmatrix} 0 & t_2\beta_2 & t_1\beta_1 & 0 & t_1\beta_1^* & 0 \\ t_2\beta_2^* & 0 & 0 & t_1\beta_1^* & 0 & t_1\beta_1 \\ t_1\beta_1^* & 0 & 0 & t_2\beta_2 & t_1\beta_1 & 0 \\ 0 & t_1\beta_1 & t_2\beta_2^* & 0 & 0 & t_1\beta_1^* \\ t_1\beta_1 & 0 & t_1\beta_1^* & 0 & 0 & t_2\beta_2 \\ 0 & t_1\beta_1^* & 0 & t_1\beta_1 & t_2\beta_2^* & 0 \end{pmatrix}, \tag{1}$$

where

$$\beta_1=2\cos(\frac{3}{2}k_x a)\cdot\exp(i\frac{\sqrt{3}}{2}k_y a)+\exp(-i\sqrt{3}k_y a),$$

$$\beta_2=1+2\cos(\frac{\sqrt{3}}{2}k_y a)\cdot\exp(-i\frac{3}{2}k_x a)\,. \tag{2}$$

Here $\mathbf{k}=(k_x,k_y)$ is the Bloch wavevector, and a is the distance between the two sublattices. The on-site energy is set to zero. Now we block-diagonalize $H(\mathbf{k})$ using the angular momentum basis as defined in Eq. (2) in the main text through a unitary transform $\tilde{H}(\mathbf{k})=U^{-1}H(\mathbf{k})U$:

$$\begin{aligned}\tilde{H}(\mathbf{k})=&\begin{pmatrix} -\mathrm{Re}(t_1\beta_1)+\sqrt{3}\,\mathrm{Im}(t_1\beta_1) & t_2\beta_2 \\ t_2\beta_2^* & -\mathrm{Re}(t_1\beta_1)-\sqrt{3}\,\mathrm{Im}(t_1\beta_1)\end{pmatrix}\oplus\begin{pmatrix} 2\,\mathrm{Re}(t_1\beta_1) & t_2\beta_2 \\ t_2\beta_2^* & 2\,\mathrm{Re}(t_1\beta_1)\end{pmatrix}\\ &\oplus\begin{pmatrix} -\mathrm{Re}(t_1\beta_1)-\sqrt{3}\,\mathrm{Im}(t_1\beta_1) & t_2\beta_2 \\ t_2\beta_2^* & -\mathrm{Re}(t_1\beta_1)+\sqrt{3}\,\mathrm{Im}(t_1\beta_1)\end{pmatrix}\end{aligned}, \tag{3}$$

where

$$U=\frac{1}{\sqrt{3}}\begin{pmatrix} 1 & e^{\frac{2}{3}\pi i} & e^{-\frac{2}{3}\pi i} \\ 1 & 1 & 1 \\ 1 & e^{-\frac{2}{3}\pi i} & e^{\frac{2}{3}\pi i}\end{pmatrix}\otimes\begin{pmatrix} 1 & 0 \\ 0 & 1\end{pmatrix}. \tag{4}$$

We can see that the original Hamiltonian is now block-diagonalized with three 2×2 blocks, and each represents a sector with a different angular momentum. Written in the Pauli matrix form, we can then obtain Eq. (3) in the main text. There is no coupling between different angular momentum sectors in this tight-binding model.

Supplementary Figure 1 shows the band structure of this tight-binding model with $t_1 = -0.5$ and $t_2 = -1$, which are similar to the band structures shown in Figs. 2(a) and 2(b) in the text. For *m*=0, these two bands are degenerate at the K point and from a Dirac cone. For *m*=1, the angular-momentum-orbital coupling term creates the band gap. The band structure for $m = -1$ is the same as that for *m*=1.

Supplementary Note 2: Band structure and transmission spectra in transmission line networks

The frame work for calculating the band structure and transmission spectra for transmission line network has been discussed in Supplementary Refs. [1-4]. For completeness, we illustrate the details of the mathematics with two elementary examples for both the band structure and transmission spectra.

We first consider a one dimensional periodic system as shown in Supplementary Fig. 2(a) as an example to demonstrate the calculation of band structures. There are two nodes C and D in each unit cell (marked by the red box). Inside the unit cell, node C is connected with node D by two transmission lines of the same length l_1. There is one transmission line with length l_2 connecting the unit cells. The lattice constant is l_0. The voltages of C and D are denoted by ψ_C and ψ_D. Applying the Bloch boundary condition, the voltage of another node D which is at the left side of the node C is written as $\psi_D \exp(-ik_x l_0)$, where k_x is the Bloch wave vector along the x direction. Using Eq. (6) in the main text, we can write the network equation for node C:

$$\left[-2\coth(gl_1)-\coth(gl_2)\right]\psi_C+\left[\frac{2}{\sinh(gl_1)}+\frac{1}{\sinh(gl_2)}\cdot\exp(-ik_x l_0)\right]\psi_D=0. \quad (5)$$

Similarly, the network equation for node D can be written as:

$$\left[-2\coth(gl_1)-\coth(gl_2)\right]\psi_D+\left[\frac{2}{\sinh(gl_1)}+\frac{1}{\sinh(gl_2)}\cdot\exp(ik_x l_0)\right]\psi_C=0. \quad (6)$$

Combining Eq. (5) and Eq. (6), the equations can be represented in the matrix form:

$$\begin{pmatrix} -2\coth(gl_1)-\coth(gl_2) & \frac{2}{\sinh(gl_1)}+\frac{1}{\sinh(gl_2)}\cdot\exp(-ik_x l_0) \\ \frac{2}{\sinh(gl_1)}+\frac{1}{\sinh(gl_2)}\cdot\exp(ik_x l_0) & -2\coth(gl_1)-\coth(gl_2) \end{pmatrix}\cdot\begin{pmatrix}\psi_C\\ \psi_D\end{pmatrix}=0. \quad (7)$$

The determinant of the coupling matrix on the left hand side should be zero for an eigenmode, which then gives us the band structure and the null space gives the eigenstate. For each Bloch vector k_x, there exists a set of frequencies where the determinant is zero. For a network with a finite size, such as the systems considered in Fig. 3, one only needs to write down the coupling equations among all the nodes and the determinant of the coupling matrix being zero gives the energy levels and corresponding eigenstates.

For an open system, additional nodes should be added corresponding to the inputs and outputs. An example with one input and one output is shown in Supplementary Fig. 2(b). There are two nodes C and D in the system and two additional nodes 'in' and 'out' are added. The two transmission lines between node C and D have the same length l_1. The length of the transmission line connecting node

‘in’ (‘out’) and C (D) is set as l_2. For node C and D, we can write their network equations respectively:

$$\left[-2\coth(gl_1)-\coth(gl_2)\right]\psi_C+\frac{2}{\sinh(gl_1)}\psi_D+\frac{1}{\sinh(gl_2)}\psi_{in}=0,$$

$$\frac{2}{\sinh(gl_1)}\psi_C+\left[-2\coth(gl_1)-\coth(gl_2)\right]\psi_D+\frac{1}{\sinh(gl_2)}\psi_{out}=0. \tag{8}$$

There are four additional equations for the incoming and outgoing waves:

$$\begin{gathered}\psi_{in}-r=1,\\ \psi_C-r\cdot\exp(-gl_2)=\exp(gl_2),\\ \psi_D-t=0,\\ \psi_{out}-t\cdot\exp(gl_2)=0,\end{gathered} \tag{9}$$

where r and t are the reflection and transmission coefficients, respectively. Here we have set the amplitude of the incoming signal as 1. Combining Eqs. (8) and (9), we can get the matrix form:

$$\begin{pmatrix}-2\coth(gl_1)-\coth(gl_2) & 2/\sinh(gl_1) & 1/\sinh(gl_2) & 0 & 0 & 0\\ 2/\sinh(gl_1) & -2\coth(gl_1)-\coth(gl_2) & 0 & 0 & 1/\sinh(gl_2) & 0\\ 0 & 0 & 1 & -1 & 0 & 0\\ 1 & 0 & 0 & -\exp(-gl_2) & 0 & 0\\ 0 & 1 & 0 & 0 & 0 & -1\\ 0 & 0 & 0 & 0 & 1 & -\exp(gl_2)\end{pmatrix}\cdot\begin{pmatrix}\psi_C\\ \psi_D\\ \psi_{in}\\ r\\ \psi_{out}\\ t\end{pmatrix}=\begin{pmatrix}0\\ 0\\ 1\\ \exp(gl_2)\\ 0\\ 0\end{pmatrix} \tag{10}$$

This equation is in the form of $M\psi=\eta$. Then all the elements in ψ can be obtained by $\psi=M^{-1}\eta$.

Supplementary Note 3: Simulated robust edge state transport

To verify the robustness of the edge states, we numerically simulate the edge state field patterns for the m=1 sector in a 7×7 sample with different scattering configurations as shown in Supplementary Figs. 3(a)-(c). Here the simulations are performed by solving Eq. (6) in the main text. Since the voltages are the same for the same position in all three layers, we plot the field pattern for only one layer. Intrinsic loss is also incorporated into the simulation here, which is why the amplitude of edge states attenuates along the propagation direction. In this simulation, the loss rate is obtained by fitting the experimental data (see Supplementary Fig. 5) and is fitted to be $L\cong 338\cdot f^{-0.6123}$, where L is in meters (m) and f is in MHz.

The signal is incident from the node marked by an arrow on the left boundary and the working frequency is set to 32.7 MHz, which is inside the nontrivial band gap. We consider three different configurations. In Supplementary Fig. 3(a), we consider a finite-size sample of the original system. In Supplementary Figs. 3(b) and 3(c), we add additional defect-induced scattering. In Supplementary Fig. 3(b), we remove all the interlayer hopping within one unit cell as highlighted in red at the upper edge. In Supplementary Fig. 3(c), we connect an additional, 2.06 m long cable (red line) to the nodes at the upper edge. Note that all the defects introduced in Supplementary Figs. 3(a-c) preserve the angular momentum. The field patterns are plotted in the right panel. We can see that, in all of the configurations above, the edge waves pass through the defect and continue to propagate forward without back-scattering.

The defects discussed above all preserve the angular momentum. Now we proceed to discuss the case when the defect does not preserve angular momentum. In this case, the defect will scatter the edge states into other subspace with different values of angular momentum.

The network we consider is a 7×7 sample as shown in Supplementary Fig. 4(a). The angular momentum non-preserving defect is introduced by removing all the interlayer cables between layer 1 and layer 2 in the red unit cells. (The interlayer cables between other layers are kept intact.) Due to the presence of this defect, the state at any node in principal contains all the angular momentum components and is written as:

$$\left|\varphi\right\rangle = c_1\left|m_1\right\rangle + c_0\left|m_0\right\rangle + c_{-1}\left|m_{-1}\right\rangle, \tag{11}$$

where the $\left|m_1\right\rangle$, $\left|m_0\right\rangle$ and $\left|m_{-1}\right\rangle$ are eigenstates with angular momentum m=1, m=0 and m=-1 respectively. The c_1, c_0 and c_{-1} are the corresponding coefficients of different angular momentum. Meanwhile, any state can also be represented in real space as:

$$\left|\varphi\right\rangle = s_1\left|j_1\right\rangle + s_2\left|j_2\right\rangle + s_3\left|j_3\right\rangle, \tag{12}$$

where the $|j_1\rangle$, $|j_2\rangle$ and $|j_3\rangle$ denotes states at layer 1, layer 2 and layer 3 respectively, and s_1, s_2 and s_3 are the corresponding coefficients. The relation between these two sets of coefficients can be written as:

$$\begin{pmatrix} s_1 \\ s_2 \\ s_3 \end{pmatrix} = \frac{1}{\sqrt{3}} \begin{pmatrix} 1 & 1 & 1 \\ e^{\frac{2}{3}\pi i} & 1 & e^{-\frac{2}{3}\pi i} \\ e^{-\frac{2}{3}\pi i} & 1 & e^{\frac{2}{3}\pi i} \end{pmatrix} \cdot \begin{pmatrix} c_1 \\ c_0 \\ c_{-1} \end{pmatrix} . \tag{13}$$

The coefficients s_1, s_2 and s_3 is obtained by measuring the complex valued voltage of each node on different layers, and c_1, c_0 and c_{-1} can then be derived using Eq. (13). In our simulations, the signal of m=1 is incident from the left middle point marked by the black arrow shown in Supplementary Fig. 4(a). Without the defect, the pure state $|m_1\rangle$ will be excited with $|c_1|$=1, $|c_0|$=0 and $|c_{-1}|$=0. With the presence of the angular momentum non-preserved defect, fields will be scattered into other angular momentum space and hence the values of c_i will change. In Supplementary Fig. 4(b), we calculated the spectra of $|c_1|^2$, $|c_0|^2$ and $|c_{-1}|^2$ on the node marked by red in Supplementary Fig. 4(a). From the spectra, we can see that the state of m=1 is now scattered to the modes of m=0 and m=-1. We also show the field pattern of the second layer at 32.7 MHz in Supplementary Fig. 4(c). Compared with Supplementary Fig. 3(b), the edge state is now scattered into the bulk due to the angular momentum non-preserved defect and the fact that the subspace with m=0 exhibits no band gap.

Supplementary Note 4: Auxiliary experimental data

In this section, we provide auxiliary experimental data to support our conclusions. The signal enters from the middle node on the left boundary of the cluster as marked by the blue arrows.

In Supplementary Figs. 7(a) and 7(b), we measure the field patterns for m=0 modes. The wave propagates from left to right at 33.7 MHz, which is inside the bulk band (Supplementary Fig. 7(a)), and is totally reflected at 49.2 MHz, which is inside the band gap (Supplementary Fig. 7(b)). We do not observe any nontrivial edge states for the m=0 sector.

Supplementary Figures 7(c) and 7(d) show the measured field patterns at 40.3 MHz (inside the bulk band) for $m = 1$ and $m = -1$, respectively. Supplementary Figures 7(e) and 7(f) show the corresponding numerical field patterns, which agree well with the experimental results.

Supplementary Figure 8 shows the experimental (a-b) and simulated (c-d) voltage field patterns for $m = 1$ (a, c) and $m = -1$ (b, d) at 31.2 MHz, which is inside the nontrivial gap region. Unidirectional edge states are observed in the experiments and the field patterns agree strongly with the simulated ones. The propagation direction is locked to the angular momentum. The voltage decays along the transportation direction due to the intrinsic loss in the cable. Note that the field patterns of m=1 and $m = -1$ are mirror symmetric to each other with respect to the x axis.

Supplementary References

1. Zhang, Z. Q. et al. Observation of localized electromagnetic waves in three-dimensional networks of waveguides. *Phys. Rev. Lett.* **81**, 5540 (1998).
2. Li, M., Liu, Y. & Zhang, Z. Q. Photonic band structure of Sierpinski waveguide networks. *Phys. Rev. B* **61**, 16193 (2000).
3. Zhang, Z. Q. & Sheng, P. Wave localization in random networks. *Phys. Rev. B* **49**, 83 (1994).
4. Cheung, S. K., Chan, T. L., Zhang, Z. Q. & Chan, C. T. Large photonic band gaps in certain periodic and quasiperiodic networks in two and three dimensions. *Phys. Rev. B* **70**, 125104 (2004).
